# A Text-Book of Mechano-Therapy

**Published:** 1899-11

**Authors:** 


A Text-book of Mechano-Therapy. (Massage and Medical Gymnastics.) Especially prepared for the use of medical students and trained nurses. By Axel V. Grafstrom, B. Sc., M. D., late Lieutenant in the Royal Swedish Army; late House Physician City Hospital, Blackwell's Island, New York. With eleven pen-and-ink sketches by the author. Philadelphia: W. B. Saunders. 1899.

This little book will doubtless be of considerable service to the trained nurse who wishes to know more of medical gymnastics and massage than the somewhat incomplete training in these branches which most hospitals give. The author describes in detail the various movements of both massage and gymnastics and illustrates various procedures by original drawings. The application of mechano-therapy to various diseases occupies about one-third of the volume.

